# Reconstructing hammerstone size flake by flake: an experimental approach

**DOI:** 10.1098/rsif.2024.0879

**Published:** 2025-05-14

**Authors:** Li Li, Shannon P. McPherron

**Affiliations:** ^1^ The Interdisciplinary Center for Archaeology and the Evolution of Human Behaviour, University of Algarve, Faro, Portugal; ^2^ Human Origins, Max-Planck-Institute for Evolutionary Anthropology, Leipzig, Germany

**Keywords:** fracture mechanics, Hertzian cone, hammerstone, ring crack, stone tool production

## Abstract

Understanding force application in flake production is essential for reconstructing hominin behaviour, technological advancements and biomechanics. Extensive research has examined stone tool production, focusing on the intended material outcomes such as the cores, tools and flakes. Analyzing force application in this process requires knowledge of hammerstone selection and use. Despite progress made in understanding hammerstone selection and use, linking specific knapping outcomes to hammerstone use remains challenging. This difficulty stems from the complex relationship between fracture mechanics and material signatures in lithic artifacts. Key variables related to hammerstone use and their influence on flaking outcomes remain poorly understood. We draw on fracture mechanics to explore factors driving flake ring crack size—the circular region where the Hertzian cone, a feature of conchoidal flaking, intersects with the platform. Our experiment systematically examines how hammerstone size, velocity and strike angle—factors influencing strike force—affect ring crack and flake size under controlled conditions. We validate our findings with previously reported controlled and replicative experiments. Results show that flake ring crack size can estimate hammerstone size. Our findings mean that we can reconstruct the flaking process and particularly variability in the application of force at a level of detail previously unavailable.

## 1. Introduction

Stone knapping necessarily involves applying a directed force to a stone to remove a flake. This force can be applied gradually and progressively (pressure flaking) or dynamically and abruptly (percussive flaking). The latter technique dates to the origins of knapping and was by far the dominant form of flaking throughout the Paleolithic. Within percussive flaking, there are several variants including freehand percussion, passive hammer, bipolar and throwing. Each of these variants, however, involves a hammerstone striking a core, though in some cases the core is moving and in other cases, the hammerstone is moving. We also know that hammerstones contribute to the variability we see in stone assemblages [1,2].

Some common approaches to studying hammerstones include documenting their presence in knapping kits within archaeological assemblages and identifying them based on percussion traces [3–13], distinguishing between the use of hard and soft hammers [6,14–26] and reconstructing the types of percussive activities the hammerstones were involved in during different stages of reduction [3,5,6,8,11,27–32].

An effective approach for studying various aspects of hammerstone use is examining the diagnostic features observable on lithic artifacts [19,21,23,24,26,33,34]. Where possible, this approach has the advantage of linking individual flakes to the use of a particular type of hammer. For instance, flakes produced by soft hammers, such as those made of bone or copper (and sometimes limestone), are typically thinner with less prominent bulbs of percussion and lipped platforms [14,16,17,26,31,35,36]. In contrast, flakes produced by hard hammers, such as those made of quartzite, have more prominent bulbs of percussion due to the distinct Hertzian cone formation resulting from the hammer blow [36–40]. Other features, such as the presence of ring cracks on the platform and ripples of fracture propagation, are associated with the use of specific types of hammerstones [15,17,18].

Use-wear analysis on lithic artifacts is helpful in identifying the presence of hammerstones based on percussive traces and reconstructing the types of percussive activities the hammerstones were involved in [5,6,8,11,13,32]. Besides identifying the use of different types of hammers, studies have also relied on features of flakes, including the size and shape of the flakes and the presence and prominence of the bulb of percussion, to distinguish the use of different percussive techniques, namely pressure flaking and percussion flaking [23,24,37,38,41–50].

In addition to working with evidence from the lithic record to directly reconstruct the selection and use of hammerstones, studies have also focused on exploring the range of manipulative gestures associated with knapping, which involves handling hammerstones [51–60]. These studies often emphasise quantifying the movement trajectories of knappers’ upper limb joints and hammerstone during different knapping processes, as well as evaluating knappers’ understanding of the effects on stone fracture processes and their ability to predict and control the knapping outcome. While valuable for understanding the manipulative processes involved in knapping, such studies tend to consider hammerstones as associated objects rather than integral aspects of the knapping process. As a result, their findings are sometimes difficult to apply directly to understanding the archaeological record.

Despite the above-mentioned insights into hammerstone use, it is still true that hammerstones can rarely be linked to specific instances of reduction strategies represented within lithic assemblages. One possible way forward is to return to conceptually what a hammerstone does, namely the application of force. Force application itself has been looked at experimentally from various perspectives, including the properties of the hammerstones (such as hardness, mass, velocity and strike angle), core raw material and how knappers manipulate their gestures during knapping [38,47,51,52,58,60–68].

Force, however, is actually a rather complex concept and is used in different ways within the archaeological literature. Some limited research has focused on deconstructing the concept of force into measurable attributes of hammerstone use, where the term force is often used to describe the intensity of a hammer blow [23,47,69,70]. In simple terms, the hammer striking force is determined by the mass of the hammer, its striking velocity at the point of impact and the contact time from the initial impact of the hammerstone on the platform to the completion of the fracture that detaches the flake (henceforth contact time). Sometimes kinetic energy (determined by hammer mass and striking velocity) is used to measure the strength of a hammer blow, as it can be more accurately and easily measured during experiments using devices such as high-speed motion capture cameras [51,54,55].

**Figure 1.**
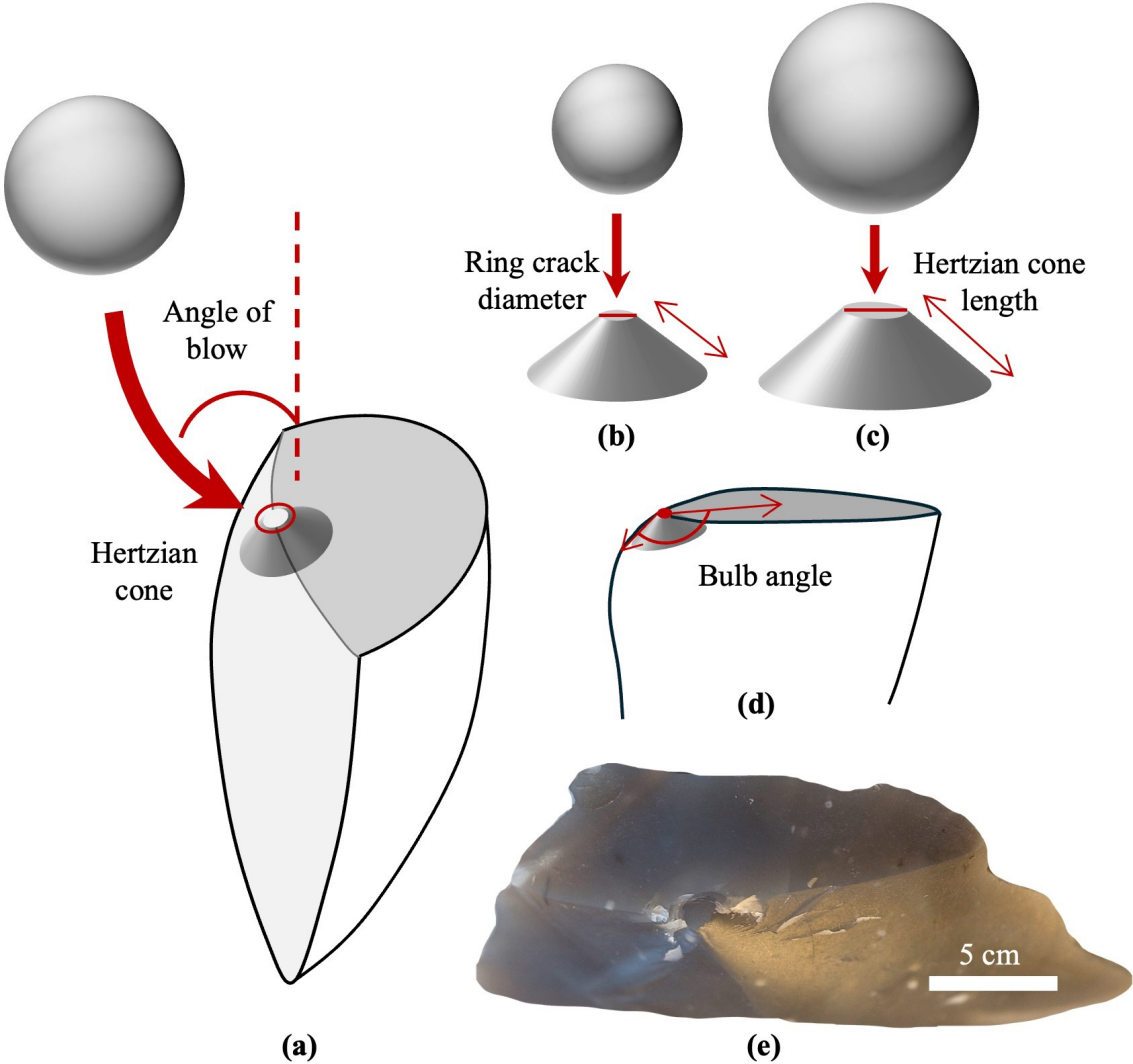
Schematic of the ring crack on a flake. (a) Illustration of the Hertzian cone (exaggerated for illustrative purposes) and its intersection with the platform to form a ring crack on a flake produced using a spherical-shaped hard hammer. The angle of blow is indicated by the red arc and is defined as the angle between the hammer strike direction and the platform’s perpendicular. (b ,c) As hammer size increases, while keeping all other factors constant, there is a corresponding increase in both the size of the ring crack and the length of the Hertzian cone crack. The diameter of the ring crack is noted with a red segment. (d) Illustration of bulb angle on a flake, defined as the angle between the protruding side of the Hertzian cone before it curves to form the bulb of percussion and the flake platform. (e) Photograph of a flint flake showing an actual ring crack clearly visible on the flake’s platform.

When describing force, it is important to consider not only its magnitude, determined by attributes such as hammer mass and impact velocity, but also the angle of blow—the angle at which the hammer strikes or force is exerted on the core for flake removal (figure 1a). The angle of blow is a key component in understanding the application of force during knapping. Previous experimental studies have shown that, under otherwise identical conditions, striking the platform at a more oblique angle produces flakes that are generally thinner and shorter [23,47]. When the hammer strikes the core perpendicularly to the platform, only a normal force is applied. However, when striking at an oblique angle, a tangential force is introduced, creating additional shear stress that aids in pulling the flake away from the core [71]. We know that knappers vary their angle of blow during knapping, such as by tilting the platform to deliver an oblique hammer strike, which assists in flake detachment [72]. Variations in the angle of blow have also been observed between expert and novice knappers [52,57,60,69]. Furthermore, evidence from the archaeological record indicates that early hominins exerted some degree of control over the angle of blow when producing flakes of different kinds [73]. The results of experimental work on knapping force application have been inconsistent. Some studies found that the force of a hammer blow does not significantly impact the flaking outcome [23,47,62]. Others show that a stronger hammer blow (e.g. using a larger hammer, a higher kinetic energy at the point of impact and applying more pressure in gripping the hammerstone) tends to produce larger flakes [37,54,60,61,74,75]. Several controlled lithic experiments conducted using mechanical apparatuses in the 1970s and 1980s generated results supporting the view that changes in hammer size significantly affect flake size by altering the impact force. Specifically, when hammer velocity is constant, a larger (heavier) hammer will produce larger flakes [37,61,75]. These findings were supported by several experimental studies conducted with human knappers [54,60,74]. However, later controlled experimental studies, some by the same authors, produced opposite results suggesting that the magnitude of force delivered by the hammer does not significantly impact the flaking outcome [47,62].

In addition to studying the overall size and shape of flakes, researchers have also examined how the size and shape of the hammerstone influence the size of the platform ring crack on a flake. The platform ring crack is the circular contact area of the Hertzian cone produced by a hard hammer blow (figure 1a,e) [18,33,34,37,45]. Drawing on fracture mechanics studies of Hertzian cone formation—a key feature in conchoidal flaking, typically resulting from direct hard hammer percussion—we know that the diameter of the platform ring crack is determined by the hammerstone’s radius, size and shape, the percussive load (force) applied, and the mechanical properties of both the hammerstone and the core material [71,76–79]. More specifically, for spherically shaped hammerstones, those with a larger radius and/or greater striking force generate larger Hertzian cones, resulting in larger ring cracks and longer edge lengths (figure 1b,c) [71,77,79–82]. In contrast, for hammerstones of other shapes, such as flat cylindrical punches, the ring crack size is determined solely by the area of the punch. A larger punch area results in a larger ring crack, while changes in striking force have no effect. For conical-shaped hammers, the size of the ring crack depends on both the striking force and the cone angle. A larger striking force and a larger cone angle both contribute to a larger ring crack [71,83].

It is important to clarify that while conchoidally fractured flakes exhibit a ring crack, fracture mechanics studies focus on the contact area between the hammer and the platform at the point of impact [83,84]. This contact area is proportionally smaller than the actual ring crack, as both theoretically and empirically demonstrated [82,84]. In this context, the contact area radius serves as a direct proxy for the ring crack radius when applying the basic formulae used in fracture mechanics to calculate contact area, as this is the primary focus of such studies.

As mentioned previously, the mechanical properties of both the hammer and the core material influence the size of the Hertzian cone. This influence is evident in attributes such as the ring crack size, the length of the cone crack path and the size of the cone angle [71,85]. In fracture mechanics studies, mechanical properties such as Young’s modulus—which describes a material’s stiffness under applied force—and Poisson’s ratio—which describes how a material deforms perpendicularly relative to applied force—are typically combined into a single modulus [71]. This combined modulus is then used to calculate attributes related to the Hertzian cone, such as the contact area size and cone crack length [71].

More specifically, based on Hertzian contact theory [71], fracture mechanics studies have derived a formula to calculate the contact area radius *a*: $a={\left(3PR/4E^{*}\right)}^{1/3}$, where *P* represents the applied load (striking force), *R* is the hammer radius and E^*^ is the combined modulus derived from the Young’s modulus and Poisson’s ratio of both the hammer and core material. Young’s modulus has a stronger influence on changes in the contact area than Poisson’s ratio; here the contact area serves as a direct proxy for the Hertzian ring crack. However, a significant challenge is that only limited fracture mechanics studies isolate individual mechanical properties to examine how changes in these properties affect Hertzian cone properties [82,86]. Moreover, in these studies, the hammer material is typically controlled, with materials like tungsten carbide or steel held constant across experiments to eliminate variability in hammer mechanical properties [77–79,87].

Previous controlled experiments have explored how both hammer materials and core materials influence flaking outcomes [23,88]. However, these studies have primarily focused on changes in hammer material—such as transitioning from ‘soft’ materials (e.g. copper, synthetic bone) to ‘hard’ materials (e.g. steel)—and their effects on flake platform type (lipped versus unlipped) or the size and shape of the resulting flakes. As for core raw materials, the focus has been on whether, under otherwise identical knapping conditions, flakes produced from different raw materials fracture in the same manner and yield flakes with similar characteristics. This is particularly relevant when compared with flakes made from glass in controlled experiments, which are sometimes criticised for lacking a clear connection to most actual archaeological materials. Although not directly related to the formation of the Hertzian cone or its size, the experimental data suggest that, within a certain range of fine-grained raw materials, these materials fracture conchoidally in a very similar manner [88]. This indicates that their Hertzian fractures follow the same Hertzian contact theory.

The premise of the work presented here is that the ring crack can be used to reconstruct the force delivered to remove a flake. Thus, based on fracture mechanics studies of Hertzian cone formation and previous experimental work [33,34,71,77,79,80], as well as the understanding that hammer striking force is determined by hammer mass, striking velocity and contact time, we predict that an increase in hammerstone size and/or velocity will result in a greater hammer striking force, generating flakes with larger ring cracks. While other factors, such as hammer velocity and material properties of the hammer and core, also impact ring crack size, hammer size is by far the most important variable in determining ring crack size. Therefore, we should be able to use ring crack size to gain insights into hammer size. This conclusion is supported not only by the controlled experiment conducted in our study and other studies [33] but also by our analysis of a replicative assemblage, where variables other than hammer size varied freely. In this analysis, we found that flake ring crack size is proportional to hammer size. Flakes produced with a larger hammer and/or faster hammer blow will also have a more prominent bulb of percussion due to the larger Hertzian cone, and will be larger in other dimensions such as length, thickness and mass. In addition to ring crack size, previous experimental work on the effect of hammerstone strike angle (the angle of blow) on flaking suggests that the shape of the ring crack will change as the angle of blow shifts from perpendicular to oblique to the platform. This change occurs because as the Hertzian cone tilts relative to the platform, the contact area of the cone and the platform surface will become more elliptical [33,44,73].

## 2. Material and methods

### 2.1. Experimental design

To test our hypothesis on the effect of hammerstone size, strike velocity and strike angle on flake ring crack size and overall flake size and shape, we conducted a controlled experimental study using a drop tower and plate glass set-up. This set-up was the same one used in Li *et al*. [38,61,62,73]. Data produced from the drop tower experiment are henceforth referred to as the drop tower dataset. The flaking apparatus is a drop tower made with a vertical stand and an adjustable supporting pole to control the drop height and, therefore, the striking velocity of the hammer. We used plate glass as the core material and steel ball bearings as hammers for flake removal. Previous studies have shown that steel ball bearings can effectively remove flakes from plate glass [33,73]. Using steel ball bearings also allows convenient control through the electromagnetic switch attached to the drop tower, which instantly releases the steel ball bearing, enabling it to drop directly onto the plate glass for flaking. Hammer strike velocity can be easily controlled by adjusting the hammer drop height. As stated above, although the absolute size of the ring crack depends on variable factors, including hammer and core material properties, the nature of the relationship between hammer size, velocity and ring crack size remains consistent regardless of changes in hammer and/or core materials. All cores were prepared to have a 65° exterior platform angle and a fixed 10 mm platform width. A more detailed description of the experimental set-up can be found in Li *et al*. [73].

We first examined the effect of hammer size on the flake’s ring crack diameter and overall size while keeping hammer velocity and the angle of blow (20°) constant. Hammer size was varied between small, with a radius of 9.5 mm and mass of 28 g, medium, with a radius of 16 mm and mass of 133 g, and large, with a radius of 45 mm and mass of 261 g. The hammers were dropped from a height of 1.18 m to maintain a consistent velocity of 4.81 m s^−1^.

Second, we examined the effect of hammer velocity on the flake’s ring crack diameter and overall size while keeping hammer size (radius = 16 mm) and the angle of blow (20°) constant. We varied the hammer velocity between low, medium and high by controlling the height at which the hammer was dropped for flake removal. Low hammer velocity means dropping the hammer from a height of 0.98 m, estimated at 4.38 m s^−1^, medium hammer velocity means a drop height of 1.18 m, estimated at 4.81 m s^−1^ and high hammer velocity means a drop height of 1.57 m, estimated at 5.55 m s^−1^. During the experiment, we found that when the hammer was dropped from heights above 1.57 m, most of the flakes produced shattered into multiple pieces, indicating that the hammer velocity exceeded a limit beyond which reliable results could be obtained. As a result, we set 1.57 m as the upper limit for the hammer drop height. Our set-up results in hammer velocities that generally fall within or close to the range exerted by human knappers, as reported in Bril *et al*. [51], where an intermediate knapper exhibits a range of 4.28–4.93 m s^−1^.

Lastly, we examined the effect of the angle of blow on the flake’s ring crack diameter and flake initiation. We varied the angle of blow across eight values (−20°, 0°, 10°, 20°, 30°, 40°, 50° and 60°), while keeping hammer size constant (radius = 16 mm). To investigate the facilitating effect of the angle of blow on flaking, we maintained constant values for hammer size and velocity (4.81 m s^−1^) and compared the range of platform depths with successful flake removal for two different angles of blow, 0° and 20°.

The independent variables varied in the experiment included hammer diameter, hammer mass, hammer velocity, hammer strike angle (the angle of blow) and platform depth. We measured hammer size by determining the steel ball bearing’s diameter with a digital caliper and measured hammer mass using a digital scale. We controlled and estimated hammer velocity based on the hammer drop height, which we measured using a laser measure from the bottom of the hammer to the surface of the core striking platform. We measured the angle of blow as the angle between the hammer strike direction and the perpendicular to the core platform using a digital goniometer, and we measured platform depth as the distance between the hammer’s point of impact and the exterior edge of the platform using a digital caliper.

We used the kinetic energy of the hammer at the point of impact, calculated based on the hammer’s mass and velocity, as a proxy for estimating the strength of the hammer blow and for comparison with forces generated by human knappers in more realistic settings [60,89]. While we acknowledge that kinetic energy at the point of impact is not equivalent to hammer strike force, our experimental set-up limits our ability to measure contact time. Hammer strike force is calculated from the change in momentum (which depends on both hammer mass and velocity) over the duration of impact (contact time). Therefore, under otherwise constant conditions, increasing the hammer mass and/or velocity will result in a greater change in momentum and, consequently, a larger impact force. However, since we cannot measure the contact time, we use the kinetic energy as a practical proxy for comparing the relative force of different hammer blows.

**Figure 2.**
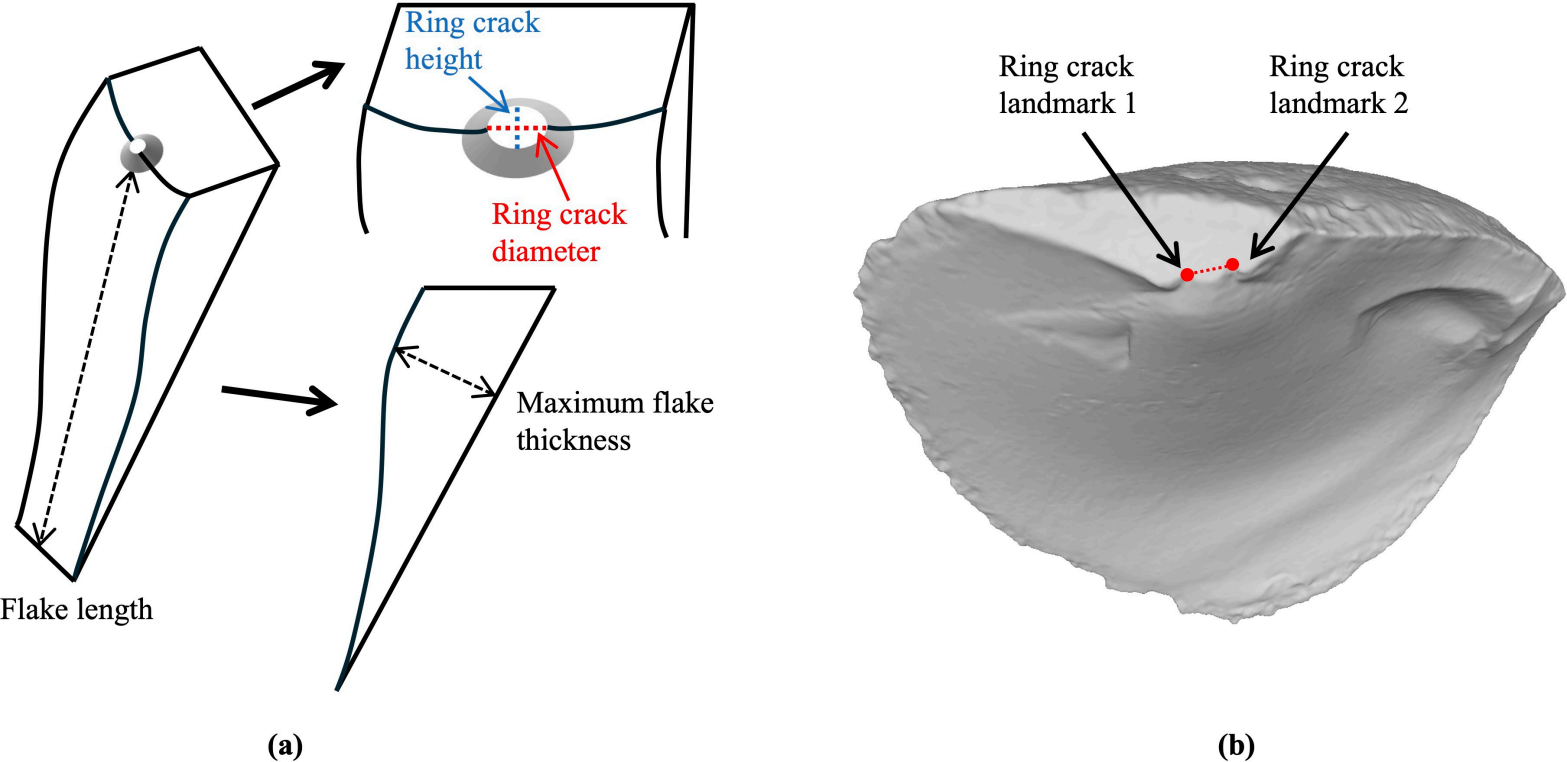
Illustration of attributes measured for the analysis. (a) Schematic of glass flakes showing the measurement of ring crack diameter (red dotted line), ring crack height (blue dotted line), flake length and maximum thickness. (b) Example of placing ring crack landmarks on a flake mesh from the Campagne dataset. The red dots represent the ring crack landmarks, and the dotted line represents the calculated ring crack diameter based on the two landmarks.

The dependent variables examined in the study included the flake’s ring crack diameter, the shape of the ring crack, flake length, thickness and mass. We measured the ring crack diameter using a digital caliper to the nearest 0.01 mm, defining it as the diameter of the circular contact area where a definitive ring crack intersects with the platform (figure 2a) [33,34]. We calculated the shape of the ring crack as the elongation ratio between its maximum perpendicular dimension (ring crack height) and its diameter (figure 2a). To ensure precise and accurate measurements, we independently measured both the ring crack diameter and height three times and used the average in our analysis. We determined flake mass with a digital scale to the nearest 0.1 g and measured flake length—defined as the distance from the point of percussion to the flake’s termination—using a digital caliper to the nearest 0.01 mm [90]. Finally, we recorded maximum flake thickness at its thickest point, including the bulb of percussion (figure 2a).

### 2.2. Additional datasets

In addition to generating our own controlled experimental dataset, we also included flake data from two other datasets. The first dataset (henceforth the Pelcin dataset) comes from Pelcin’s dissertation and provides additional information and context to the current study [33]. In the experimental studies conducted by Pelcin [33], the flakes were made from plate glass using a drop tower set-up similar to the one used in our study. Steel ball bearings were used as hammers, controlled by an electromagnet switch. Hammer sizes ranged from small (9.5 mm radius) to medium (17 mm) and large (25 mm). Pelcin tested angles of blow of 0°, 5°, 10°, 25°, 35° and 50°. The measurements we use here coming from Pelcin’s dataset, including ring crack diameter, hammer diameter, hammer mass and hammer drop height, were taken by Pelcin following the same protocol described earlier for the drop tower experiments, as published in his dissertation [33]. Pelcin did measure the angle of blow differently. He measured the angle between the hammer strike and the platform surface (rather than the surface normal). To make his data comparable to ours, we transformed Pelcin’s angle of blow by subtracting it from 90°.

The second dataset is from a series of experimental replicates of various Middle and Upper Paleolithic core reduction strategies. Details of the digital version of this dataset (henceforth the Campagne dataset) used here are reported in Archer *et al*. [91,92], and today the collection is stored in Campagne, France. The flakes were originally produced to replicate different Middle and Upper Paleolithic core reduction strategies. They are all unretouched and complete and made by skilled and experienced knappers. The knappers who produced these flakes were unaware of the intent of our study, which was to examine the influence of hammer size on flake ring crack size. This allowed us to conduct a blind test to independently verify whether the results from the experimental study could be extended to a less controlled dataset more like an archaeological dataset. The raw material used was Bergerac and Sénonien flint from southwest France. With the Campagne dataset, we test whether the flake ring crack diameter is sensitive to changes in hammer size. While we understand that raw material properties, such as Poisson’s ratio, influence ring crack size, the flakes from the Campagne dataset were made from the same raw material source, minimizing this effect. This allows us to better investigate the impact of hammer size on ring crack size. We selected 50 complete and unretouched flakes that were produced with hard hammers of different sizes with information on broad strategy and reduction sequence for analysis. All flakes from the Campagne dataset were previously scanned using an Artec surface scanner. For the study presented here, one of us (L.L.) landmarked the flake meshes to identify the two points on the interior platform edge where the flake’s ring crack intersects (figure 2b), doing so without prior knowledge of the hammer used in the reduction sequence. We then calculated the ring crack diameter as the Euclidean distance between the two landmarked points.

### 2.3. Statistical comparison

First, we examined the individual effect of each of the force delivery variables (i.e. hammer size, hammer velocity and the angle of blow) on the ring crack diameter. We ran the Kruskal–Wallis test (which makes no assumptions about the data distribution) to examine the effect of hammer mass on the ring crack diameter. Given our model and sample size, we expected that for all of the three datasets, there would be a statistically significant difference in the flake’s ring crack diameter between the different hammer size groups, with a larger and heavier hammer producing a larger ring crack diameter. For the experimental dataset, we also ran the Kruskal–Wallis test to examine the effect of hammer velocity and the angle of blow on the flake’s ring crack diameter. Similarly, we expected that both the angle of blow and hammer velocity would have a significant effect on the ring crack diameter. Post hoc analyses were performed using the Wilcoxon pairwise test to identify which specific groups of the test variables had a significant difference in ring crack diameter.

To investigate the relationship between hammer size, hammer velocity and the angle of blow on the flake’s ring crack diameter, we conducted a multiple linear regression analysis. Our goal was to determine the relative importance of each variable in predicting the ring crack diameter. We standardized each of the independent variables and the dependent variable to have a mean of zero and a standard deviation of one. This approach allowed us to compare the magnitude of the coefficients from the linear regression analysis in a more meaningful way. The standardized regression coefficient indicated the number of standard deviations the predicted value (in this case, the ring crack diameter) changes with each one standard deviation change in the independent variable (i.e. hammer size, hammer velocity and the angle of blow). By assessing the relative importance of each independent variable in predicting the ring crack diameter, we could better interpret the strength of their effects.

Second, we conducted multiple linear regression to examine the effect of the hammer strike force on the flake’s ring crack diameter and size. We used the kinetic energy, calculated from hammer mass and impact velocity at the point of impact as a proxy for measuring the force of the hammer strike. Hammer mass and impact velocities were measurable relatively straightforwardly given the experimental set-up. We standardized flake length, thickness and mass by platform depth to control for collinearity [47,61,73]. We used hammer mass and impact velocity individually as independent variables and flake dimensions as response variables. We also used both hammer mass and velocity as independent variables to examine their combined effect and significance in predicting flake size.

## 3. Results

### 3.1. Flake ring crack size and shape

**Figure 3.**
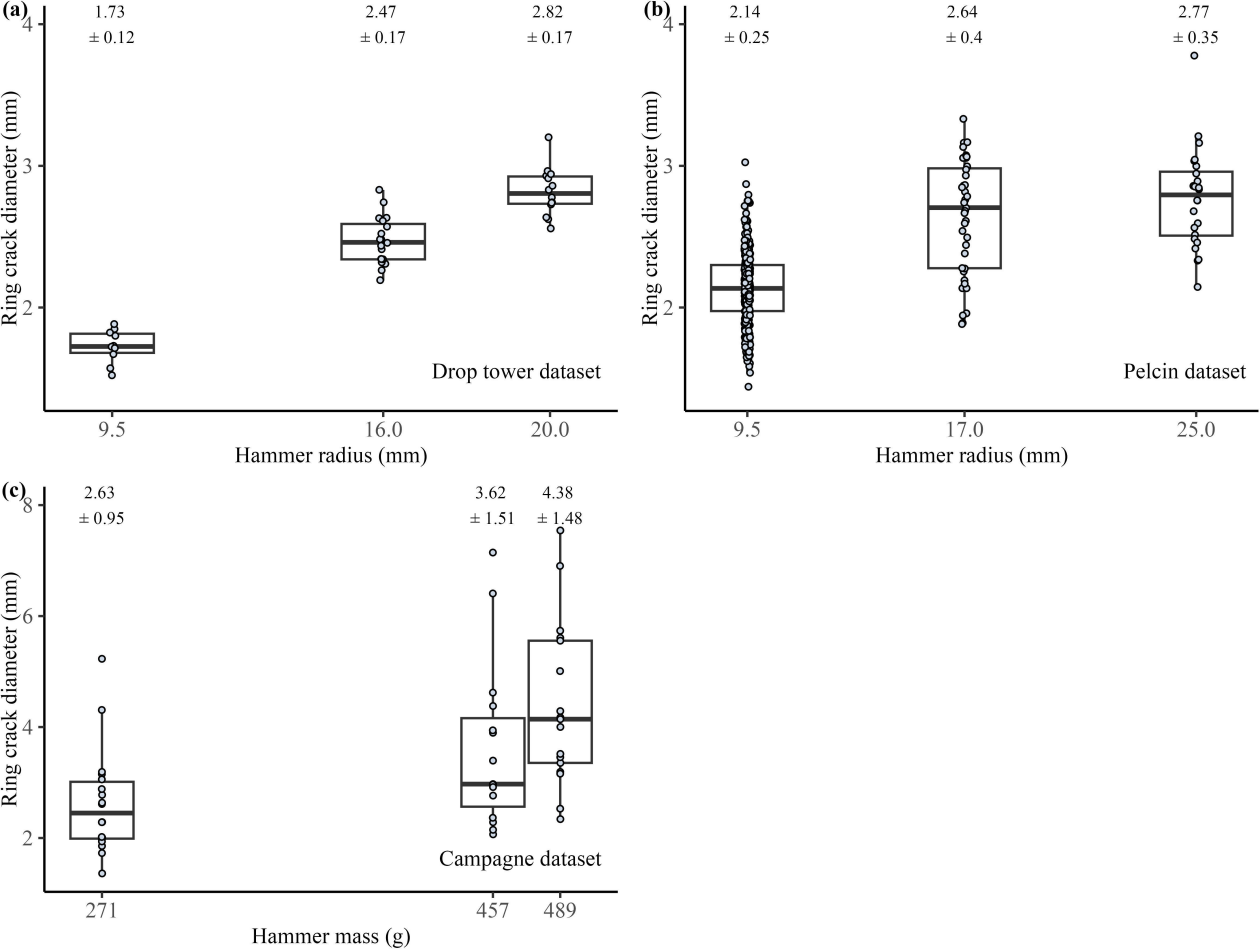
Relationship between flake ring crack diameter and hammer size. (a) Boxplot showing the relationship between flake ring crack diameter and hammer radius in the drop tower dataset. (b) Boxplot showing the relationship between flake ring crack diameter and hammer radius in the Pelcin dataset. (c) Boxplot showing the relationship between flake ring crack diameter and hammerstone mass for the Campagne dataset. In each boxplot, the box represents the interquartile range (IQR), spanning from the 25th to the 75th percentiles of ring crack diameters. The whisker lines extend to the minimum and maximum values within 1.5 times the IQR, indicating the range of data. The black line inside the box represents the median ring crack diameter. Across all datasets, we observe a significant positive correlation between hammer size (mass or radius) and flake ring crack diameter.

For the drop tower dataset, while keeping hammer velocity (4.81 m s^−1^) and the angle of blow (20°) constant, we generated flakes using three hammers with radii of 9.5 mm (*n* = 10), 16 mm (*n* = 19) and 20 mm (*n* = 14). We found that there is a significant difference in the ring crack diameter between the different hammer size groups (Kruskal–Wallis test, *H* = 32.843, *p <* 0.001, see electronic supplementary material, table S1) (figure 3a). Post hoc Wilcoxon pairwise analysis shows that the difference in flake ring crack diameter is significant across all three hammer groups (see electronic supplementary material, table S2). Furthermore, we calculated the average ranks of all values for each hammer size group. Our results show that flakes produced with the small hammer had the lowest average rank (5.5), followed by the medium hammer (8.5) and the large hammer (10.5). This pattern aligns with our experimental observation that as hammer size increases, the overall flake ring crack diameter also increases. Results from the Pelcin dataset are similar to those from the drop tower dataset (Kruskal–Wallis test, *H* = 55.771, *p* < 0.001, see electronic supplementary material, table S3). Post hoc Wilcoxon pairwise analysis shows that the difference in flake ring crack diameter is significant across all three hammer groups. For flakes generated using hammers with radii of 9.5 mm (*n* = 357), 17 mm (*n* = 39) and 25 mm (*n* = 24), when hammer velocity (4.95 m s^−1^) and the angle of blow (20°) were held constant, flakes produced by the larger and heavier hammers exhibited larger ring crack diameters (figure 3b). The average rank analysis further supports this trend, with flakes from the small hammer having the lowest rank (95.8) and those from the large hammer having the highest rank (194.9), reinforcing our observation.

For the Campagne dataset, there is a significant difference in flake ring crack diameter between the different hammer size groups (Kruskal–Wallis test, *H* = 15.407, *p <* 0.001, see electronic supplementary material, table S4). For the three hammers of different masses, 271 g (*n* = 18), 457 g (*n* = 15) and 489 g (*n* = 17), the results align with observations from the drop tower and Pelcin datasets. Specifically, flake ring crack size increases as hammer size increases (figure 3c). Post hoc Wilcoxon pairwise analysis shows that the flake ring crack produced by the lightest hammer is significantly smaller than those produced by the two larger hammers. However, there is no significant difference in flake ring crack size between the two larger hammers of masses 457 and 489 g (see electronic supplementary material, table S5).

For the drop tower dataset, while keeping the hammer size constant and varying hammer drop height (meaning varying velocity at impact), there is no significant difference in flake ring crack size between the three hammer drop height groups, where the same hammer (radius = 16 mm) was dropped from heights of 0.98, 1.18 and 1.57 m, corresponding to estimated impact velocities of 4.38, 4.81 and 5.55 m s^−1^.

**Figure 4.**
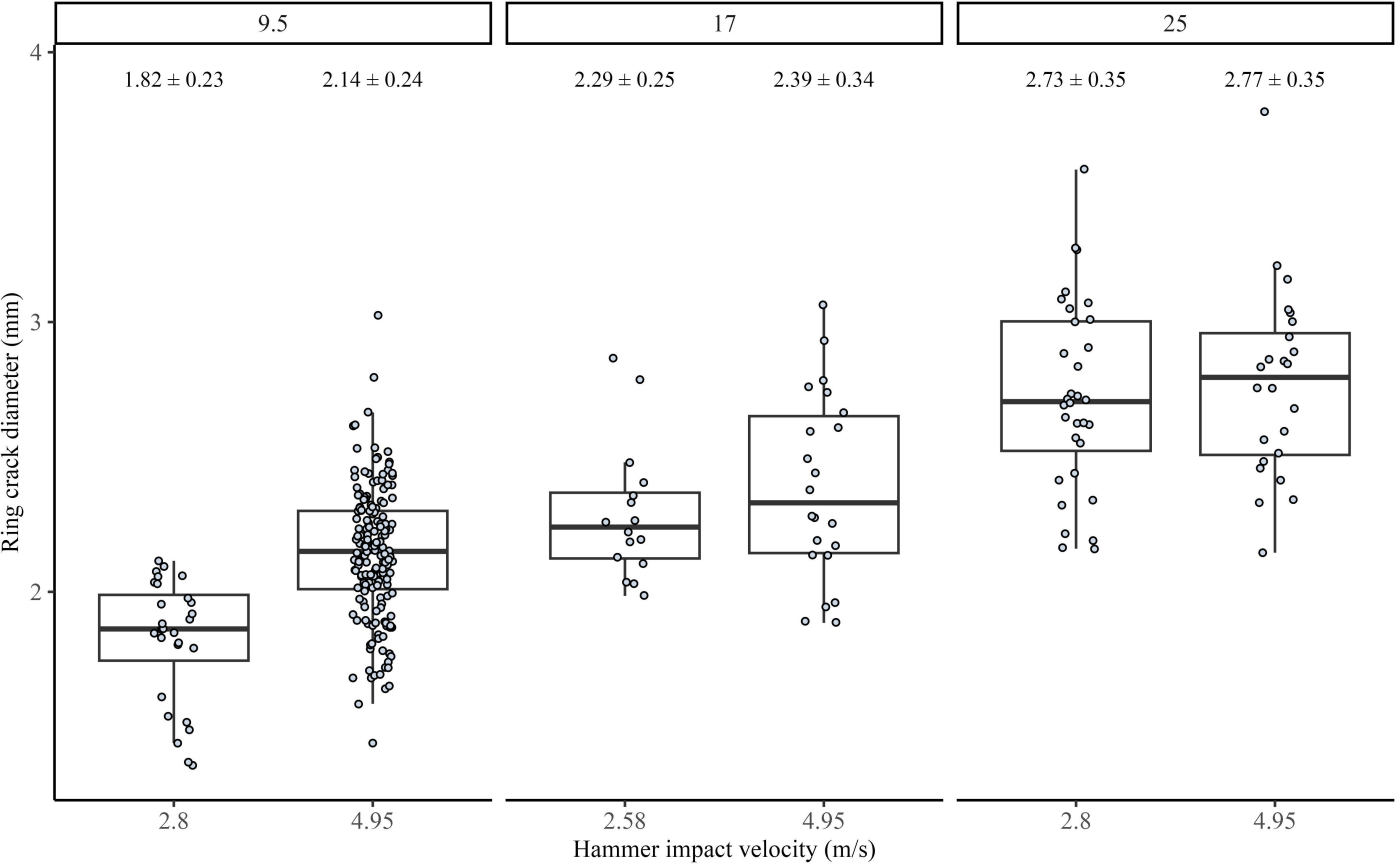
Boxplots of the Pelcin dataset showing the relationship between flake ring crack diameter and hammer impact velocity, controlled by hammer drop height. The flakes are grouped by hammer radius of 9.5, 17 and 25 mm. All flakes were produced with an angle of blow of 50°. In each boxplot, the box represents the interquartile range (IQR), spanning from the 25th to the 75th percentiles of ring crack diameters. The whisker lines extend to the minimum and maximum values within 1.5 times the IQR, indicating the range of data. The black line inside the box represents the median ring crack diameter.

For the Pelcin dataset, however, we find that while keeping the angle of blow constant, within each hammer mass group, hammers dropped from a higher height, hence with a higher impact velocity, consistently produced flakes with larger ring crack diameters (figure 4). The ring crack diameter measurements in the Pelcin dataset follow the same protocols as those used in our experimental dataset. Kruskal–Wallis tests also show that the difference in ring crack diameter between each drop height within a hammer size group is significant for all three hammer size groups. We calculated the effect size for each hammer size group to assess the variability in ring crack diameter explained by differences in hammer velocity. The effect sizes are 0.281, 0.077 and 0.043 for the hammer size groups of 9.5, 16 and 25 mm. These results suggest that, compared with flakes from the 9.5 mm hammer group, the difference in ring crack diameter for flakes from the 16 and 25 mm groups is statistically significant but relatively small in practical terms. Further analysis using linear regression, which models ring crack diameter based on hammer radius and impact velocity (ring crack diameter ~ hammer radius + hammer velocity, with hammer velocity controlled by hammer drop height), shows that both hammer radius and impact velocity have a significant impact on ring crack diameter. More specifically, hammer radius has a stronger effect on ring crack diameter than hammer impact velocity, as indicated by the coefficients (table 1).

**Table 1.** Results of the linear regression predicting flake ring crack diameter using hammer mass and impact velocity (controlled by hammer drop height) from the Pelcin dataset. All flakes were produced with an angle of blow of 50°. We standardized the independent variables to facilitate easier comparison of the coefficients.

	estimated coefficients	s.e.	*t*-value	*p-*value
intercept	0	0.042	0.00	1
hammer radius (scaled)	0.749	0.046	16.23	0
hammer velocity (scaled)	0.271	0.046	4.92	0
adjusted *R*^2^: 0.472				

**Figure 5.**
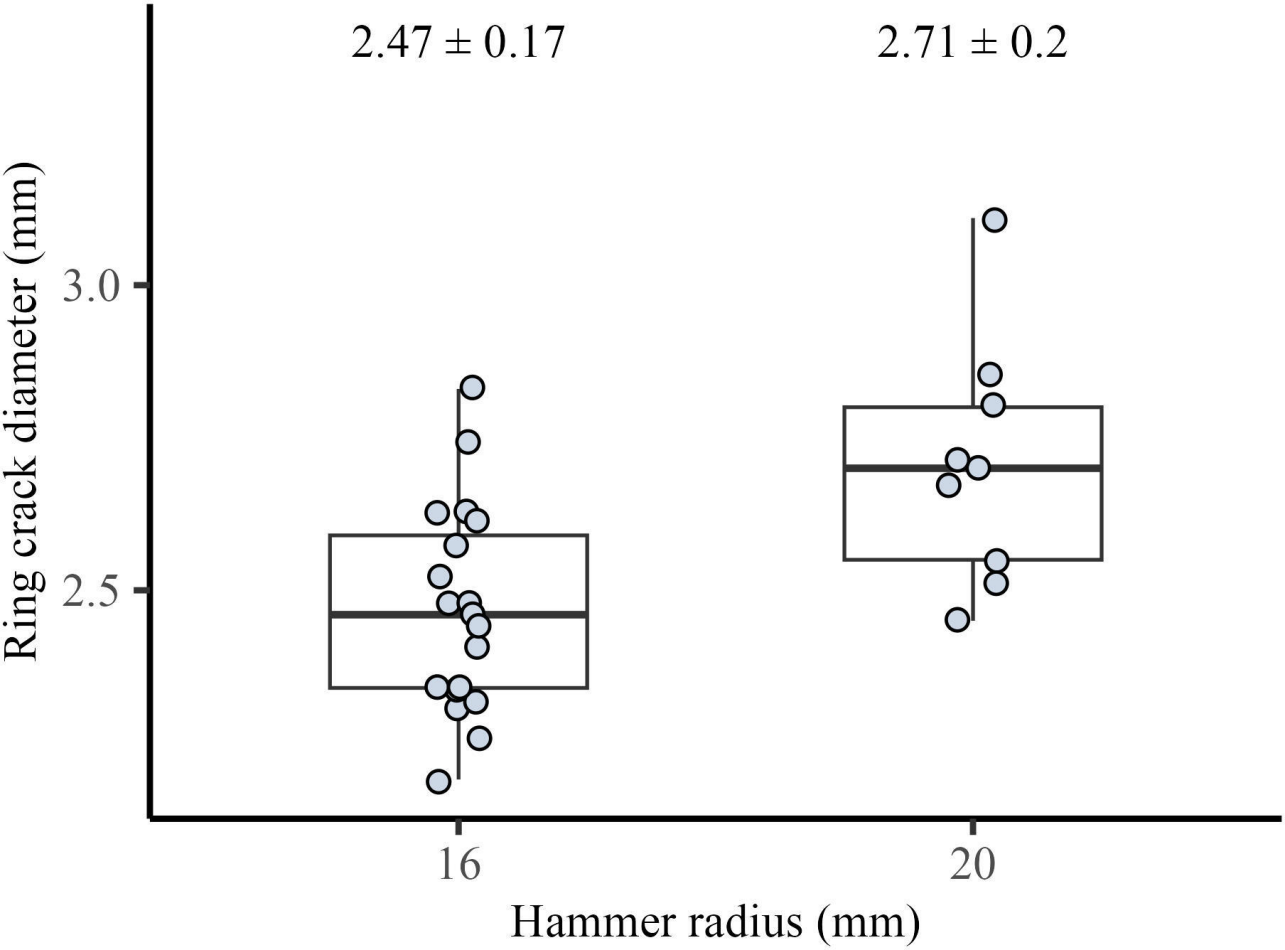
Boxplot of the drop tower dataset showing the relationship between hammer radius and flake ring crack diameter when the hammer’s kinetic energy at the point of impact is kept constant. All flakes were produced with a 20° angle of blow.

For the drop tower dataset, by varying hammer mass and drop height, we maintained the hammer’s kinetic energy at a constant 1.54 J. We dropped the medium-sized hammer from a height of 1.18 m and the large hammer from a height of 0.6 m. While keeping the hammer’s kinetic energy at the point of impact constant, flakes produced with the large hammer have a significantly larger ring crack size than those produced by the medium-sized hammer (Kruskal–Wallis test, *H* = 7.47, *p =* 0.006, figure 5).

**Figure 6.**
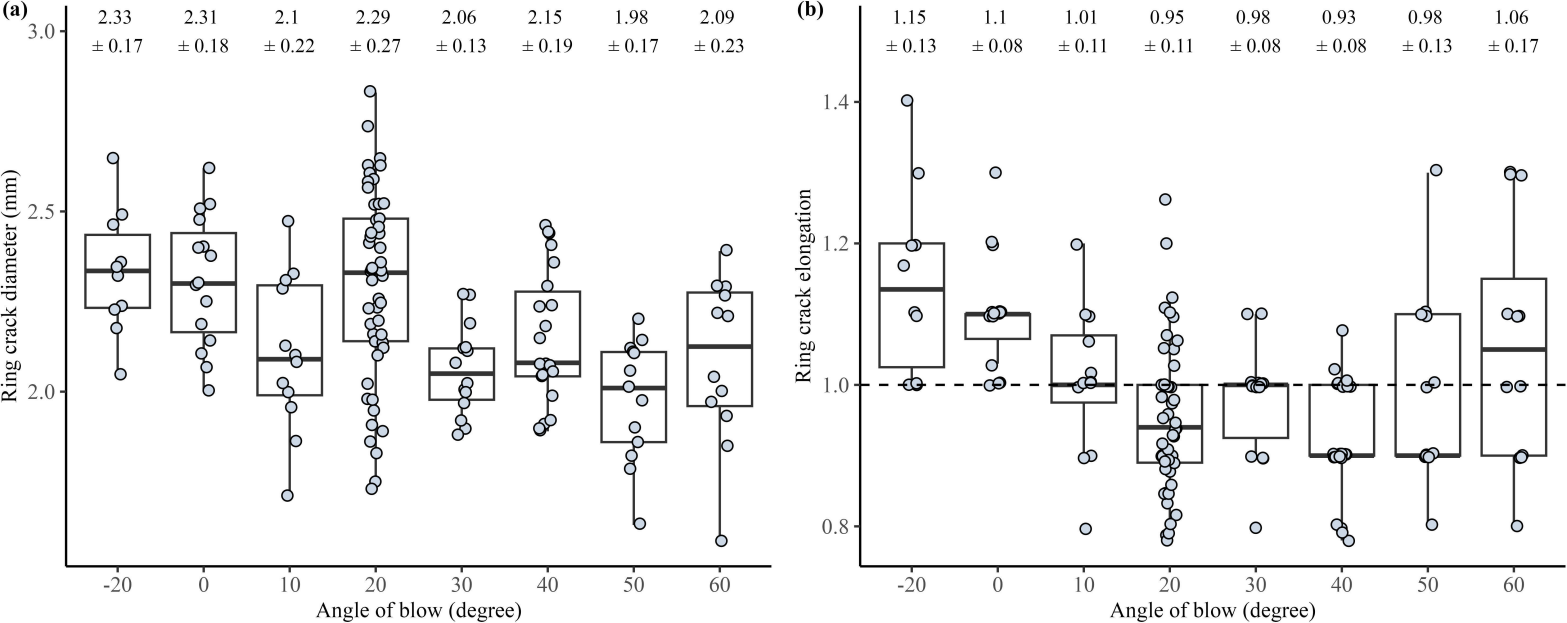
Boxplots of the drop tower dataset showing the relationship between flake ring crack size and shape and the angle of blow. (a) Boxplots showing the relationship between the angle of blow and flake ring crack diameter. (b) Boxplots showing the relationship between the angle of blow and flake ring crack elongation ratio (ring crack height/ring crack diameter). All flakes were produced using the 133 g hammer.

For the drop tower dataset, we observe a significant difference in ring crack diameter between the angle of blow groups (Kruskal–Wallis test, *H* = 35.14, *p <* 0.001, figure 6a). Overall, the more direct angle of blow (i.e. smaller angles of blow) produces flakes with a larger ring crack diameter and the more oblique angles of blow (i.e. the bigger angles of blow) produce flakes with a smaller ring crack diameter. A closer examination of the post hoc analysis following the Kruskal–Wallis test shows that there is no significant difference in ring crack diameter between flakes struck with blow angles of 20° and above. In addition, there is also a significant difference in the ring crack shape (the ring crack height to diameter ratio) between the different angle of blow groups (Kruskal–Wallis test, *H* = 43.29, *p <* 0.001, figure 6b). As shown in figure 6b, from −20° to 20° of angle of blow, the ring crack becomes less elongated and then remains fairly constant. The data appear to show an increase in elongation again at 50° and 60°. For the Pelcin dataset, however, we find no significant difference in the ring crack diameter between the angle of blow groups (see electronic supplementary material, figure S1).

For the drop tower dataset, results from the linear regression analysis examining the effect of hammer mass and angle of blow on the ring crack diameter (ring crack diameter ~ hammer mass + angle of blow) show that both hammer mass and angle of blow are significant predictors of the ring crack diameter (table 2). As the hammer mass increases, the ring crack diameter also increases. Conversely, as the angle of blow becomes more oblique relative to the platform (i.e. increases its value), the ring crack diameter decreases. Specifically, hammer mass has a strong positive effect on ring crack diameter, while the angle of blow has a modest yet significant negative effect on ring crack diameter. Hammer velocity does not have a significant effect on ring crack diameter.

**Table 2.** Results of the linear regression analysis predicting flake ring crack diameter based on hammer radius, hammer impact velocity, and the angle of blow for the drop tower dataset. The regression model includes all three variables as predictors. We standardized the independent variables to facilitate easier comparison of the coefficients.

	estimated coefficients	s.e.	*t*-value	*p-*value
intercept	0.027	0.038	0.712	0.478
hammer radius (scaled)	0.935	0.044	21.189	0
angle of blow (scaled)	−0.109	0.038	−2.865	0.005
hammer velocity (scaled)	0.080	0.044	1.831	0.07
adjusted *R*^2^: 0.84				

### 3.2. Flake size

**Figure 7.**
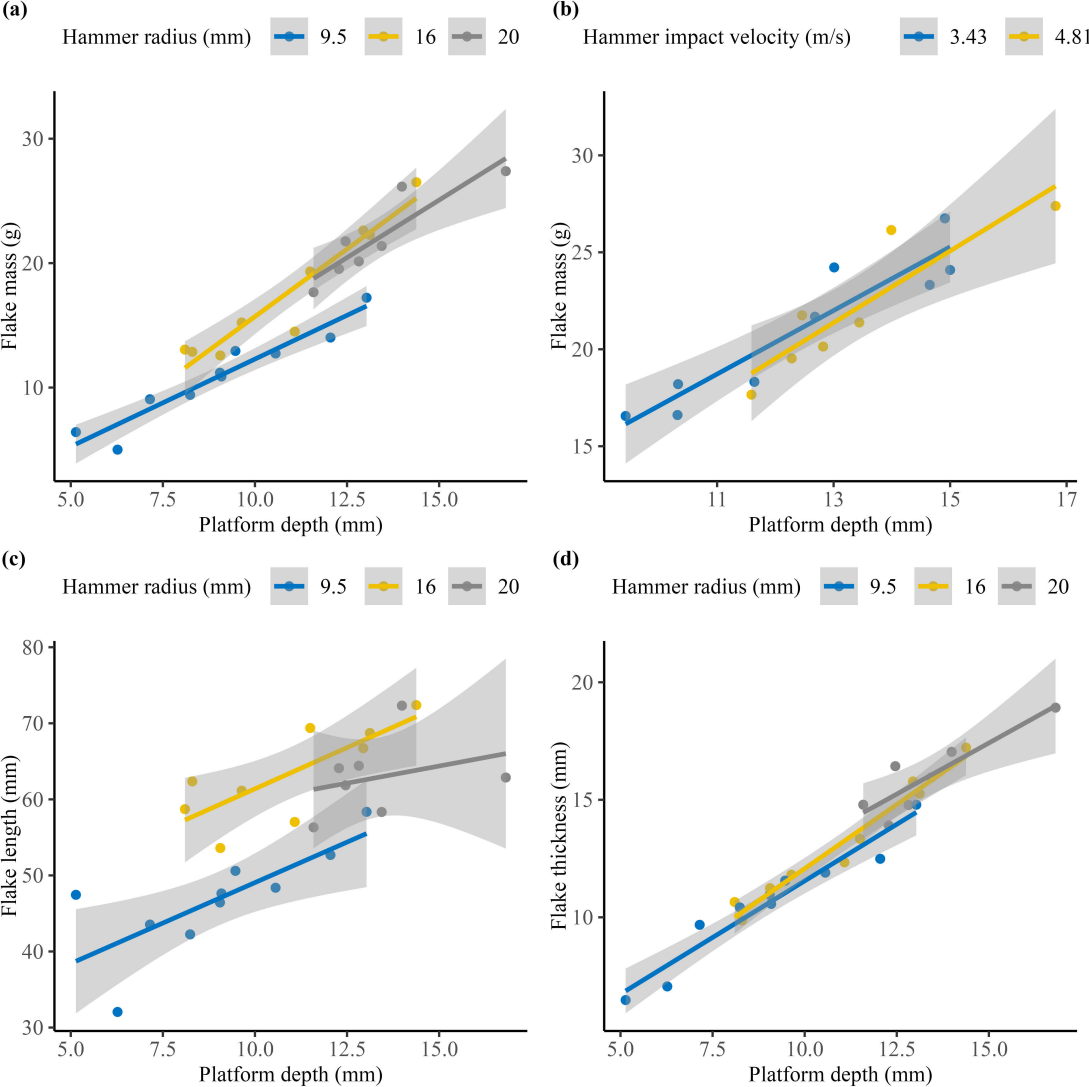
Scatter plots of the drop tower data showing the relationships between platform depth and different flake metrics. (a) Relationship between platform depth and flake mass, all hammers were dropped from the same height of 1.18 m. (b) Relationship between platform depth and flake mass, where the same hammer (radius = 16 mm) was dropped from two different heights. (c) Relationship between platform depth and flake length, with all hammers dropped from the same height of 1.18 m. (d) Relationship between platform depth and maximum flake thickness, with all hammers dropped from the same height of 1.18 m.

We first examined the effect of hammer mass and velocity separately on flake size. For the drop tower dataset, when we held the hammer velocity at the point of impact constant at approximately 4.81 m s^−1^, the medium-sized hammer and the large-sized hammer produced notably heavier flakes for a given platform depth compared with the small-sized hammer (figure 7a). However, we find no discernible difference in flake mass between the medium and large hammers. A closer examination of the data shows that the increase in flake mass resulting from the use of a larger hammer is primarily attributed to the increase in flake length and thickness. As the size of the hammer increases, longer and thicker flakes are produced (figure 7c,d). On the other hand, when we kept the hammer size constant, there is no significant difference in the mass of the flakes produced at the two different velocities (figure 7b).

For the drop tower dataset, we also used platform depth and flake ring crack diameter as predictors to model flake mass. In the model, we kept the angle of blow at 20° due to its known effect on flake mass from various studies [25,47,78]. While platform depth is a significant predictor of flake mass, we found that the addition of flake ring crack diameter significantly improves the model’s performance, increasing the *R*^2^ value from 0.885 to 0.924 (table 3).

**Table 3.** Results of the linear regression analysis predicting flake mass using platform depth (PD) and flake ring crack diameter (RCD) from the drop tower dataset.

model	predictor	estimate	s.e.	*t*-value	*p*-value	adjusted *R*²
flake mass ~ PD	PD	0.942	0.051	18.800	0	0.885
flake mass ~ PD + RCD	PD	0.858	0.499	17.210	0	0.925
	RCD	0.217	0.497	4.374	0	

### 3.3. The facilitating effect of the angle of blow on flake initiation

**Figure 8.**
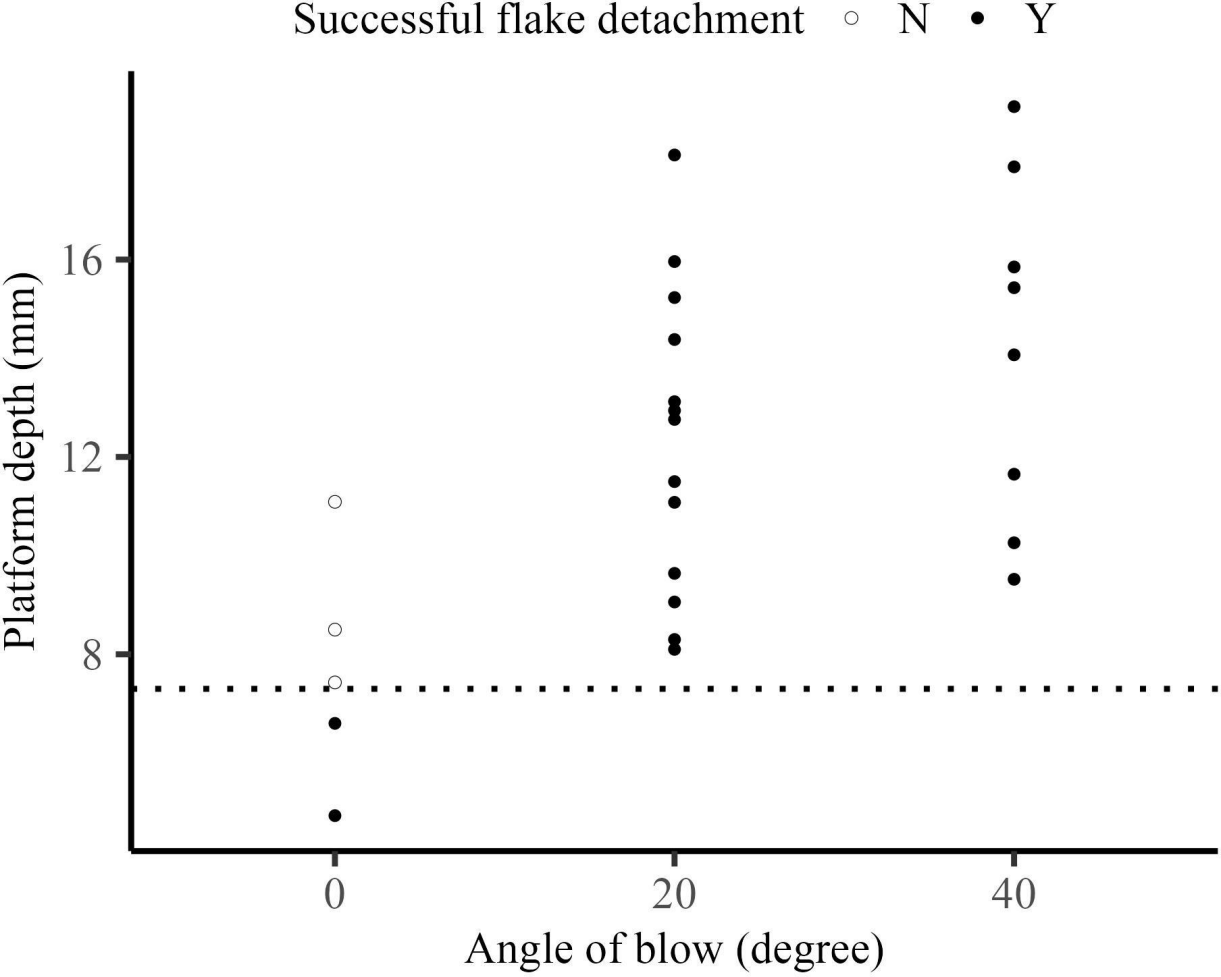
Range of platform depths with the same hammer size and velocity for the drop tower dataset. Solid black points indicate successful flake initiation; open circle points indicate the formation of only a Hertzian cone without flake initiation.

We observed a facilitating effect of the angle of the blow on flake initiation. Our experimental results show that, with constant hammer size and velocity, a more oblique angle of blow increases the upper limit of platform depth and facilitates the removal of larger flakes. As shown in figure 8, when using the medium hammer dropped from a height of 1.18 m with a zero angle of blow, we could not produce flakes beyond a platform depth of 8 mm. However, under the same conditions, flakes made with more oblique angles of blow (20° and 40°) exhibited a much wider range of platform depths, with flakes still being produced at platform depths of 16 mm and greater.

## 4. Discussion

The size and material type of hammerstones used are key aspects in understanding the knapping strategies and biomechanics of hominins. In this study, based on knowledge from previous lithic studies and fracture mechanics research, especially those related to Hertzian cone formation [71,78–80,93], we hypothesize that changes in hammerstone size could be detected in individual flakes by examining the ring crack size. Our results show that changes in flake ring crack diameter can reflect changes in hammerstone size. Although other attributes such as hammer impact velocity and the angle of blow also affect the size of the flake ring crack diameter, hammerstone size has the strongest effect. This positive relationship between hammerstone size and flake ring crack diameter is evident across different datasets and raw material types (soda-lime glass and flint).

The relationship between flake ring crack diameter and hammer size is explained by the fracture mechanics of Hertzian cone formation. Under otherwise identical conditions, spherically shaped hammers with a larger radius and a larger striking force will result in a larger ring crack. The size of the hammerstone and striking force are key attributes in determining the size of the contact area between the hammer and the platform during impact, which corresponds to the ring crack observed on flakes produced through hard hammer percussion.

As discussed previously, the hammer striking force is determined by hammer mass, impact velocity and contact time from the point of impact to flake detachment (or the end of the fracture travel). Given the limitations of the drop tower set-up, we simplified the scenario by varying only hammer size and impact velocity. We also used hammer mass and impact velocity to estimate the kinetic energy of the hammer at the point of impact, approximating the force of the hammer strike. For the drop tower dataset, we found that hammer size has the most significant and measurable effect on flake ring crack diameter, a finding that is also supported by the Pelcin dataset. The blind test on the Campagne flint dataset further supports our findings from the two controlled experimental datasets on glass. Even in the less controlled setting of the Campagne dataset, we observed significant differences in flake ring crack diameter when using hammers of different sizes. This strongly suggests that this finding can be applied to the archaeological record.

According to fracture mechanics studies of Hertzian cone formation [71,77,79], the size of the ring crack is determined by factors including hammer radius, impact force and the mechanical properties of both the hammer and the core. However, it should be noted that the experimental set-up did not allow us to separate hammer size into mass and radius as two independent variables. As a result, a hammer with a larger radius will always be heavier, contributing to a larger impact force, which is determined by the hammer mass, impact velocity and contact time. Given that contact time could not be controlled with the experimental set-up, we instead varied hammer mass and impact velocity to control the kinetic energy of the hammer.

Results from our drop tower experiment show that dropping the same hammer from different heights, hence with different impact velocities, does not have a significant effect on the flake ring crack diameter. However, results from the Pelcin dataset show that hammer velocity indeed has a significant effect on flake ring crack size; with the same hammer, a higher impact velocity leads to a larger kinetic energy and possibly impact force (assuming no significant change in contact time). This observation aligns with fracture mechanics studies, where both hammer radius and impact force are factors determining ring crack size. The discrepancy in the effect of hammer velocity between our drop tower data and the Pelcin dataset can be explained by the difference in hammer velocity in our drop tower experiment (a 1.38 m s^−1^ difference) not being large enough to generate a significant difference in ring crack size, compared with the Pelcin experiment (a 2.15 m s^−1^ difference).

We also varied hammer mass and impact velocity between two hammers (weighing 133 and 261 g) so that both hammers bore the same kinetic energy at the point of impact. Results show that the larger hammer produced flakes with bigger ring crack diameters, highlighting the significant effect of hammer size, specifically its radius, not mass, on flake ring crack size. Although we were unable to separate hammer mass and radius, this result clearly demonstrates that it is the radius of the hammer that exerts the significant impact on flake ring crack diameter.

Besides hammer size and impact velocity, the angle of blow also influences the size, shape and formation of the flake’s ring crack. As the angle of blow changes—e.g. from a position perpendicular to the platform to a more oblique angle—a tangential force is introduced, causing the Hertzian cone to tilt towards the platform. Consequently, the contact area between the Hertzian cone and the flake’s platform changes shape, as the platform now intersects the cone at an angle rather than along a perfectly horizontal plane.

Results from our drop tower experiments show a modest yet significant negative relationship between flake ring crack diameter and the angle of blow. We find that, as the angle of blow becomes more oblique relative to the platform, the flake ring crack diameter decreases slightly. This decreasing trend in ring crack diameter stops when the angle of blow reaches 20°. This observation aligns partially with findings from a previous study by Li *et al*. [73], which noted that the flake bulb angle (figure 1d)—the angle between the extruding side of the Hertzian cone and the flake platform—stabilises around 40°. This stabilisation of ring crack elongation suggests that, at an angle of blow of approximately 20°, the inner side of the Hertzian cone has fully transferred onto the platform, causing the size and shape of the ring crack to no longer change. However, this pattern of ring crack diameter decreasing as the angle of blow becomes more oblique was not observed in the Pelcin dataset.

There is also a significant relationship between flake ring crack shape and angle of blow in the drop tower dataset. The ring crack becomes less elongated as the angle of blow becomes more oblique, with the elongation ratio stabilising at approximately 30°. Similarly to the observation that ring crack diameter holds steady at an angle of blow around 40°, the stabilisation of the elongation ratio could be explained by the Hertzian cone being fully transferred onto the platform, causing the ring crack shape to cease changing.

Interestingly, the elongation ratio of the ring crack is approximately 1, indicating a nearly perfect circle at an angle of blow of 10° (ratio of 1.01), rather than at 0° (ratio of 0.95), where the angle of blow is perpendicular to the platform. This suggests that, contrary to theoretical expectations where the Hertzian cone should create a round circle at 0°, unforeseen variables not controlled in the experimental set-up, such as the hammer and core materials and the specifics of flake detachment, may influence these results. In fracture mechanics studies, the fracture usually stops after the formation of the Hertzian cone, whereas detaching a flake from a core surpasses this stage and introduces additional complexities not typically studied.

Although results from both the drop tower and Pelcin datasets show that hammer size is not the only variable determining flake ring crack diameter, it has the strongest effect compared with other variables such as hammer velocity and the angle of blow. For the drop tower dataset, the linear regression model predicting ring crack diameter using hammer mass and the angle of blow shows that while the angle of blow has a modest yet significant effect, hammer mass has a strong and significant effect. For the Pelcin dataset, the linear regression model predicting ring crack diameter using hammer mass, drop height (as a proxy for velocity), and the angle of blow shows that, compared with hammer velocity, hammer mass has a much stronger effect on ring crack diameter. Both linear regression models emphasise the strong significant effect of hammer size on flake ring crack diameter compared with other attributes such as hammer velocity and the angle of blow. This indicates that changes in hammer size can be more easily detected by examining flake ring crack size. This conclusion is also supported by the results of the blind test on the Campagne dataset, where despite varying reduction strategies, we observed a significant difference in flake ring crack diameter between hammerstones of different sizes.

However, we note that the controlled experiments were conducted exclusively on glass using steel ball bearings as hammers, whereas our blind test involved flint flakes produced with rock hammers. From relevant fracture mechanics studies on ring crack size, we know that, in addition to hammer size and striking force, the mechanical properties of both the hammer and core material also influence the size of the ring crack [71,77,79,81,82]. This means that while the relationships shown here will still hold true, the absolute ring crack size cannot be directly compared across different raw material types. However, empirical data on ring crack size across different raw materials and hammer types (soft or hard) remain limited, so it is difficult to know the impact on size these differences make [82].

Using published formulae based on Hertzian contact theory from fracture mechanics literature ($a=(3PR/4E^{*}{)}^{1/3}$) [71], we can estimate the impact of changes in core and hammer material properties on flake ring crack size. This provides a ballpark estimate of the effect of raw material properties on ring crack size within a range while holding hammer material constant. It is clear that for specifically shaped hammers, when both hammer material and shape are controlled and core raw materials are also controlled, the only factors affecting ring crack size are hammer striking force and hammer radius. Since both the glass flakes from controlled experiments and the flint flakes from replicative experiments were analyzed exclusively for Hertzian fractures, it is reasonable to generalize the results observed in the glass experiments to the flint flakes and study hammer size based on flake ring crack size.

From the Hertzian contact theory, we deduce that when using the same hammer, ring cracks are larger on softer rocks with higher Poisson’s ratios and lower Young’s moduli (i.e. materials that deform more perpendicular to the applied force and are less stiff [71]), while harder rocks produce smaller ring cracks. Conversely, when keeping the core material constant and changing the hammer type (while maintaining the same size and shape), softer hammers produce flakes with larger ring cracks compared with harder hammers. It should also be noted that only hard hammers were used in the Campagne dataset, so hammer material does not interfere with our observations of factors contributing to changes in ring crack size on flakes.

As such, it is important to continue investigating the effects of hammer and raw material properties, such as Young’s modulus and Poisson’s ratio, on ring crack size. By incorporating values for Young’s modulus and Poisson’s ratio of the hammer and core material, we can refine estimates derived from Hertzian contact theory and compare them with empirical data. Understanding the extent of variation in raw material properties is crucial for establishing a framework to compare ring crack sizes across different raw material types, providing deeper insights into the use and selection of hammerstones. In addition to flake ring crack diameter, we also observe from the drop tower dataset that flakes produced with the two larger and heavier hammers are longer, thicker and therefore heavier than flakes produced with the small hammer (figure 7*a*,*c*,*d*). For the drop tower dataset, the linear regression model predicting flake mass using platform depth and hammer mass shows that besides platform depth, which has been repeatedly proven in studies to be one of the primary factors in determining flake size [47,61,62], hammer radius is also a significant predictor, although not as strong as the effect of platform depth. The addition of hammer radius to the linear regression model significantly improves its performance. Here it should be noted that there is a direct positive correlation between hammer mass and radius. Hammer radius is also a significant predictor of flake mass, just like hammer mass. We further substituted hammer mass with flake ring crack diameter, which also proves to be a significant predictor for flake mass. Based on our experiments and the fracture mechanics of the Hertzian cone, flakes produced with a larger hammer show both a larger ring crack and a longer Hertzian cone crack, resulting in an overall larger Hertzian cone [71,77–80]. This larger Hertzian cone can result in a flake with a larger bulb, contributing to increased thickness and length that allows fractures to travel. Our observation aligns with findings from several previous studies [38,61].

It should also be noted that while the two larger hammers produced significantly heavier flakes than the small hammer, there is no difference in flake mass and dimensions between the medium and large hammers. This could be because the medium hammer already generated enough force to remove a flake with maximum size given the platform depth. Increasing the force beyond this threshold will not result in a larger flake, even though it will result in a larger ring crack, while below the threshold, an increase in hammer size will result in an increase in striking force, which will then lead to an increase in flake size. Our observation of the relationship between hammer size and flake mass can be simplified as within the threshold for generating flakes of maximum size given the platform configuration, hitting the core with a bigger hammer will result in a bigger flake.

This conclusion may appear contradictory to findings in other studies [47], where a load cell measured the hammer strike force for flake removal. In that study, the hammer applied pressure until the flake was removed, potentially always applying the maximum force needed, resulting in flakes reaching maximum dimensions given the platform configuration. In contrast, in our drop tower dataset, we controlled hammer impact force by varying hammer mass and velocity. In a realistic knapping scenario using two hammers—one small and one large—even if a harder or faster blow from the small hammer is used to generate more striking force, the larger hammer with a lighter or slower tap could still generate enough force to match or exceed the impact from the small hammer. Using the larger hammer would result in a flake with a larger ring crack and, consequently, a larger flake, irrespective of hammer impact velocity.

Results of our drop tower experiment also demonstrate the facilitating effect of the angle of blow on flake initiation, as supported by fracture mechanics literature [71]. We find that, compared with a direct angle of blow, an oblique angle of blow significantly increases the range of flake platform depths that can result in successful flake removal. That is, while using the same hammer and striking velocity, striking the core at an oblique angle can produce flakes that are much larger than those produced by striking the core at a direct angle. This is because an oblique hammer strike introduces a shear load in addition to the normal load perpendicular to the platform, which helps initiate the crack for flaking. Studies have also documented that knappers tend to strike the platform at an oblique angle when making flakes with large platforms [16], taking advantage of the facilitating effect of the angle of blow.

Despite other factors, we find that hammer size has the most significant effect on flake ring crack diameter. As a result, flake ring crack diameter can be used as a proxy for measuring changes in hammerstone size, as also suggested in other studies [15,17,18]. To further study knapping strategies from the lithic record, we should combine flake ring crack diameter and flake bulb angle, which has been proven to be an effective measure for the angle of blow [73,94], to reconstruct past knappers’ choices of hammerstone size and hammer strike angle from a more holistic perspective.

Together these results suggest that insights into hard hammer selection can be made from the archaeological record by measuring ring crack diameter and examining how this varies with other variables such as stage of reduction, flake size and flake technology. With actual hammerstones, as opposed to perfectly spherical metal ball bearings used in the experiments, we can expect ‘effective’ hammer radius (as measured by the size of the impact surface) to vary across a single hammerstone and to vary through the use life of a hammerstone as it becomes worn and facetted. The Campagne dataset, however, suggests that the relationship between hammer size and ring crack size is sufficiently strong as to remain measurable despite these other factors. The next step is to investigate whether we can detect if ring crack size varies systematically within or across archaeological assemblages to better reconstruct past knapping behaviour. Our findings in this study help contextualize patterns that could be observed in ring crack size, potentially in relation to other flake metrics and technical attributes that may reveal aspects of knapping behaviour in the archaeological record.

## 5. Conclusions

Results from our study indicate that flake ring crack diameter can serve as a proxy for hammer size, albeit with some limitations. While we acknowledge that hammer impact velocity and strike angle also influence flake ring crack size, it is evident from our experiments and the study conducted by Pelcin that hammer size has an unparalleled significant effect on flake ring crack diameter [33]. Even in the Campagne dataset that represents a less controlled setting, we were able to detect changes in hammer size using flake ring crack diameter as a proxy. Although we could not separate hammer mass and radius to study their individual effects on flake formation, we nonetheless demonstrated the prominent effect of hammer size (specifically hammer radius) on both flake ring crack size and overall flake dimensions. Furthermore, integrating flake ring crack size and bulb angle (which is a proxy for hammer strike angle) allows us not only to identify the size of the hammerstone but also to infer the strike angle used by knappers. Given the facilitating effect of the angle of blow on flake initiation, measuring flake ring crack diameter and bulb angle has the potential to reconstruct how past knappers adjusted their hammer strike angle according to the size of the hammerstone used when producing flakes of different sizes. Additionally, analyzing variations in both flake ring crack diameter and bulb angle allows us to study the consistency of knappers’ choices regarding hammer size and strike angle, which could reflect their level of knapping expertise.

Despite its potential in understanding hominin tool production behaviour, it should be noted that the flake ring crack is a small feature that may not always be observable on various flakes made on various raw materials. Additionally, factors such as hammer geometry, hammer material and core material also influence the size and shape of the ring crack, requiring further investigation. Our study highlights the critical role of integrating fracture mechanics into experimental lithic studies, as emphasised by prior research [38,39,68,73,95]. This approach enables us to generate findings grounded in fundamental principles of physics, with direct relevance to archaeological interpretations.

## Data Availability

All data generated or analyzed during this study are included in this published article and its Supplementary Information files and are also available in the OSF repository [96]. Supplementary material is available online [97].
